# Practice of Medicine

**Published:** 1860-01

**Authors:** 


### PRACTICE OF MEDICINE.

THE PATHOLOGY AND TREATMENT OF GALL-STONES. By *Dr. Thudichum.*—The author introduced his subject by stating that those who engaged in post-mortem examinations of the human body had, not rarely, an opportunity afforded them of examining concretions in the gall-bladder or gall-ducts, which for several centuries had engaged the attention of the learned and roused the wonder of the curious. To either, it must be a subject of astonishment that a large number of solid hard bodies should be met with filling almost the entire cavity of a receptacle destined to hold a mild and innocuous fluid, and that yet there should have been no symptoms of their presence during the life of the individual who carried those concretions in his biliary organs. To many, however, gall-stones proved by no means mild and innocuous, but, by frequent and painful effects, only too sensibly reminded them of their existence. These painful and sometimes fatal attacks the physician was called upon to relieve, to heal, or, as the case might be, to prevent. Like the morbid anatomist, the physician had to deal with the consequences of a disease which was itself not the object of his immediate attention. Both investigators might be compared to the geologist, whose inquiry into the history of an outburnt volcano had to be carried on upon the ashes, the lava, and other rocky products, and upon the large features of their conformation; it was impossible for him to observe their direct genesis—the fire that produced them was long since extinct. An inquiry into the pathology of gall-stones was mostly an inquiry into causes so remote and obscure that the difficulties encountered in such a proceeding ranked among the greatest which medical science had to battle with. Accordingly, our positive knowledge of those causes amounted to very little or nothing; surmise had taken the lead, and had assigned various causes, which the author mentioned at length, but which, on a stringent scrutiny, he said, must stand aside as incompetent. Nevertheless, it was his opinion that so soon as the chemistry of the liver and bile was well understood, we should be in a position to approach the problem from both sides—during life and after death—by physiological research and experiment, as well as by the anatomical and chemical examination of the dead body.

The author then related a case, being one of several in which Mr. Holmes, of St. George's Hospital, had kindly afforded him an opportunity of examining the liver and bile of deceased persons. The notes relative to the case had been given from the register of St. George's Hospital, by Dr. G. G. Rogers. They showed the subject to have been a married woman of sixty years of age, who was received into the hospital, under the care of Dr. Pitman, suffering from valvular disease of the heart, hemorrhagic infractus of both lungs, and dropsy. She had died within a week from the time of her admission.

On examining the bile in the gall-bladder, the author found it to consist of a homogeneous fluid, containing little coloring matter in solution; but a large amount of brownish-yellow coloring matter, together with many crystals of cholesterine, were suspended in it. It was analyzed, and the result of the analysis, which was given in outline, was that it did not contain a trace of biliary acid.

The gall-stones were about sixteen in number. On dividing the largest one into halves, the author found a large nucleus of brown pulpy matter, which could be easily removed with the point of a knife, or washed away with a brisk stream of water from a so-called wash-bottle. When collected in a white china dish, the matter appeared to be composed of thread-like films of different diameters, some a quarter of an inch long; some shorter pieces were one-sixteenth of an inch in thickness. All were cylindrical, as if molded in tubes; many had branches, and others divided dichotomically. The thinnest portions had a diffuse, broom-like end, as if the matter had not had time or quality to solidify in the tubular form, or as if it had solidified in a bag-like enlargement of the cylinder in which the rest of the cast was molded. The matter composing these productions was granular, without a trace of crystallization of any kind, was purely yellow in the thinnest branches, but became darker brown the thicker the forms grew. Forty or fifty medical gentlemen, to whom the author had an opportunity of submitting both the specimens and drawings of them, had borne witness to the accuracy of the representations. All of them agreed with him that these peculiar formations could be nothing else than *casts of the biliary ducts*. They were so fragile that the mere weight of a thin glass cover, as used for microscopical preparations, was sufficient to crush the thinner ones. When shaken in the watch-glasses in which the author kept them moistened with glycerin, the mere friction of one against the other would damage and disintegrate the most characteristic feature. About half the number of gall-stones, and among them some large-sized ones, although containing a good brown nucleus, yet did not admit of the separation of characteristic casts; but among the *débris* some fragments of casts could be distinguished with ease and certainty. From their extreme delicacy, and a variety of other circumstances, the author thought it a mere chance whether such casts, once formed, should be preserved, or destroyed by mixture with crystallized cholesterine, from which they could not be mechanically separated. They might hence occur more frequently in gall-stones, or otherwise, than we are able to find them.

The material of the casts was not chemically homogeneous. A yellow portion was extracted; another portion, probably bilifulvine, remained. Some had a peculiarly ragged or variably projecting outline, which made the author examine for cylindrical epithelial cells; but however great the probability that such cells might adhere to the circumference, or enter into the body of the casts, being epithelial proper to the biliary ducts, certain it was that no such formations could be identified.

Dr. Thudichum then animadverted upon the numerous reflections suggested by this observation. He was of opinion, he said, that true bilious attacks, and cases of acute, so called, idiopathic jaundice, might hereafter find an explanation by the discovery of a real and material obstruction of the passages of the bile by formations similar to those which he had described. After referring to a case given by Frerichs, which seemed to present a feature in point, he mentioned the branched calculi, and also the branched gall-stones found by Dufresne, in the finer ramifications of the biliary ducts, as presenting, perhaps, some analogy to the casts described.

The author then discussed at length the possible or probable circumstances which may produce gall-stones. The main cause appeared to him to be the decomposition in the gall-bladder of the solvent of cholesterine—taurocholic acid.

Having related several cases in which the author had found gall-stones in the gall-bladder or liver after death, and having recapitulated the various descriptions of gall-stones, he remarked upon the symptoms produced by these concretions during life. He concluded his remarks upon the passages of gall-stones through the biliary ducts, with the statement that he had not been able to find a case on record of a person who, during life, suffered from well-authenticated attacks of the passages of gall-stones, and was, after death, found not to harbor some concretion or other in his biliary passages.

After referring to the general termination of cases of all kinds, and relating some in point from his own experience, Dr. Thudichum detailed a case which he had had an opportunity of treating for several years and watching to its conclusion, and which he considered to be an appropriate introduction to the question of treatment. The case showed that gall-stones might exist in the gall-bladder during forty years, produce recurrent attacks, and yet, with prudent living and medical assistance, the patient might attain the age of eighty. The aged patient took the celebrated mixture of turpentine and ether with so much apparent benefit, that during four years following the last of several attacks of passing gall-stones, he allowed few days to pass without taking from half a drachm to a drachm of the mixture. After his death, the gall-stones were found very soft and pulpy, by which observation the theoretical value of the ether and turpentine mixture assumed a sort of empirical confirmation, although, on strict scrutiny, the direct solvent action of these substances could not well be understood.

For the symptomatic treatment of the passage of gall-stones, the author said we had, as heretofore, to rely mainly upon opium, which was sometimes better borne in the form of pills than in that of tincture. An overdose of this drug was to be guarded against, as severe narcotism sometimes followed large doses of opium, when the pain, which caused it to be given, suddenly subsided, from local causes. A case which had happened in Ireland some years ago, the author thought, suggested caution. Patients were, however, more apt to take excessive doses of opium on their own account than the practitioner was likely to prescribe them.

After some remarks upon the dietary and hygienic rules to be observed by gall-stone patients, the author suggested that in some appropriate cases an operation for the removal of gall-stones through the abdominal walls should become a subject of consideration for surgeons. When the cause and origin of gall-stones were a little better known, they might be prevented, and to that time he looked forward with confidence and hope.

Mr. Harrison was happy to bear out one of the statements of the author by a case of his own. He exhibited the gall-bladder of a male patient who died of pulmonary consumption, which was closely packed with numerous small and large cholesterine calculi, incrusted by some white phosphate of lime. There had been no symptoms arising from these concretions during life. He also adverted to a second case of his, which terminated fatally, in which the prolonged jaundice and ultimate death had been caused by the arrest of a single large calculus in the common duct.

Mr. Canton had found gall-stones repeatedly after death in very fat subjects, and thought there might be some connection between the obesity and these concretions. The coincidence of the atheromatous condition of the arteries with gall-stones, as mentioned in the first case of the author, was also in accordance with his own experience. He inquired whether the author had made analyses of blood as to the quantity of cholesterine contained in it.

Dr. Richardson having alluded to the effects of the loss of bile by a biliary fistula in animals, which he thought was contra-indicating the proposed operation, referred to points of diagnosis. He had in two cases been able to verify the diagnosis by Dr. Cockle, of gall-stones in the gall-bladder, by the sound produced by these concretions when brought into sudden contact by percussion. He also mentioned a case of his own, in which he found sixteen calculi disposed in the liver; and concluded with some interesting remarks upon the connection of gall-stones with gout.

Mr. Gay had treated two cases in which gall-stones were discharged through the abdominal parietes by the spontaneous act of nature. They made their exit near the umbilicus. It was remarkable that in neither case were they accompanied by biliary matter, nor followed by the establishment of biliary fistulæ.

Mr. Ross had seen three cases of extreme deposit of fat, in which gall-stones were discovered after death. He thought the observation, by the author, of casts of the biliary ducts, a most important discovery, and considered his paper a turning-point for future inquiries.

Dr. Hare animadverted upon the common coincidence of gall-stones with pulmonary phthisis, and said that excess of fat could not in these cases be any etiological consideration. The dilatations in the gall-ducts which had been mentioned in one of the author's cases he considered to be cavities left by the softening of tubercles. They were also found in phthisis, but ordinarily only in tuberculous subjects below sixteen years of age. He did not agree with Rokitansky as to their being enlargements of the biliary ducts, as he had never observed any communications with such ducts.

Dr. Smith inquired as to the rationale of the dietary rules laid down by the author. He combated the notion of an etiological relation between obesity and gall-stones, particularly upon the basis of his own experience in phthisis.

The President remarked upon the operation which the author had hinted at, and thought that there were many difficulties in its way. He should like to hear from physicians the number, nature, and prospects of such cases in which the operation for extracting gall-stones could be thought of. The operation would be impossible in cases where the calculus was closely embraced by the bladder. But, from his knowledge, he thought it not impossible that cases fit for operative relief might present themselves; and in cases of distended gall-bladder, (which might occur with calculi,) an operation such as the author had mentioned had actually been performed with success.

Dr. Thudichum, in reply, expressed his thanks for the kindness and attention which the Society had manifested. The cases and opinions brought forward by the gentlemen who took part in the discussion had been eminently interesting to all and instructive to himself. He could not reply seriatim to all observations, and would therefore only say that, according to his statistical researches, no habit or temperament of body could be accused of favoring the production of gall-stones. The fat and the lean, the new-born infant and the octogenarian, were alike subject to the complaint. As a general rule, he thought gall-stones the sequel of an acute process which produced insoluble coloring matter, perchance casts of the biliary ducts, upon which the cholesterine was deposited. But whether the cholesterine merely deposited upon the coloring matter as it would upon any other foreign body, or whether its precipitation was also produced by and an essential symptom of the first disorder, he hoped to ascertain by future observation and experiments upon animals. Cholesterine was a product of the activity of the liver, and, like all biliary matters, not performed in the blood. But the study of the blood was nevertheless of importance, and he had himself made analysis of it in relation to the question of gall-stones.—(*London Lancet*, October 22, 1859.)

